# The effect of the four pharmacological pillars of heart failure on haemoglobin level

**DOI:** 10.1097/MS9.0000000000001773

**Published:** 2024-01-29

**Authors:** Darshan Hullon, Erfan Taherifard, Taif Haitham Al-Saraireh

**Affiliations:** a MercyOne Clinton Medical Center, Clinton, IA; b Hematology Research Center, Shiraz University of Medical Sciences, Shiraz, Iran; c Prince Hamzah Hospital, Amman, Jordan

**Keywords:** Adrenergic Beta-Antagonists, anaemia, angiotensin receptor antagonists, angiotensin-converting enzyme inhibitors, heart failure, mineralocorticoid receptor antagonists, sodium-glucose transporter 2 inhibitors

## Abstract

Anaemia, a condition characterized by low levels of haemoglobin, is frequently observed in patients with heart failure (HF). Guideline-directed medical therapy improves HF outcomes by using medications like beta blockers, angiotensin-converting enzyme inhibitors, and angiotensin receptor blockers, along with mineralocorticoid receptor antagonists and sodium-glucose cotransporter 2 inhibitors. In this study, we aimed to review the pathophysiology of anaemia in patients with HF and present the current evidence regarding the relationship between the main recommended medications for these patients and haemoglobin levels. The authors conducted a comprehensive search in the medical literature for relevant original clinical articles in which the four pharmacological pillars of HF were given to the patients; we, then, assessed whether the association of use of these medications and haemoglobin level or development of anaemia was provided. These common medications have been shown in the literature that may exacerbate or ameliorate anaemia. Besides, it has been shown that even in the case that they result in the development of anaemia, their use is associated with positive effects that outweigh this potential harm. The literature also suggests that among patients receiving medications with negative effects on the level of haemoglobin, there was no difference in the rate of mortality between anaemic and non-anaemic patients when both were on treatment for anaemia; this point highlights the importance of the detection and treatment of anaemia in these patients. Further research is needed to explore these relationships and identify additional strategies to mitigate the risk of anaemia in this population.

## Introduction

Highlights

- Anaemia is a common condition observed in patients with heart failure.
- Beta blockers, angiotensin-converting enzyme inhibitors, angiotensin receptor blockers, mineralocorticoid receptor antagonists, and sodium-glucose cotransporter 2 inhibitors are among guideline-directed medical therapy highly recommended for patients with heart failure.
- Evidence from the literature suggests that the use of guideline-directed medical therapy, even though improves the outcomes of patients with heart failure, may exacerbate or ameliorate anaemia.
- The use of angiotensin-converting enzyme inhibitors, and angiotensin receptor blockers was observed to be associated with a decreased level of haemoglobin.
- The use of sodium-glucose cotransporter 2 inhibitors was found to have a positive impact on the level of haemoglobin.

Anaemia is among the most common comorbid conditions that could be found in patients with heart failure (HF). It has been estimated that approximately one-third of these patients have various degrees of anaemia; the prevalence, however, ranges from less than 10–70% depending on the population studied, design of the study, definition used, severity of HF, etc.^1,2^. Studies have reported that the presence of this common comorbidity in HF patients worsens their prognosis and it is an independent predictor of mortality and morbidity^3,4^. In a recently published meta-analysis, data from more than 53 thousand patients with HF with anaemia in about 36% of them, were pooled^5^. In this study, it was revealed that having comorbid anaemia was associated with significantly higher odds of hospitalization and all-cause mortality within one year (odds ratio of 1.22 with 95% CI, of 1.0–1.58 and 1.43 with 95% CI of 1.25–1.63, respectively). Besides, it has been shown that anaemia could significantly affect the quality of life, physical functioning, and perceived general health and well-being^6–8^ and result in a higher economic burden^9^.

Literature suggests that the development of anaemia in patients with HF is multifactorial and is not completely understood. Hemodilution, state of iron deficiency, inflammation, dysregulated production of erythropoietin, or its structural alterations are among the main mechanisms that contribute to anaemia^10,11^. Evaluation of anaemia and its underlying causes is highly important^12^ and its treatment seems to exert beneficial effects not only on the symptoms of the patients but also on the prognosis^13^; In a longitudinal study on patients with chronic HF (CHF), statistical analyses showed that the rate of mortality was significantly lower for the patients whose haemoglobin level increased over the study^14^. Therefore, its management has been recommended for these patients^15,16^. Studies have also shown that medications that are prescribed for HF could result in induction or worsening of the pre-existing anaemia through several means^11,17–19^. In this study, we aimed to review the pathophysiology of anaemia in patients with HF and present the current evidence regarding the relationship between the main recommended medications for these patients and anaemia.

## Pathophysiology of anaemia in HF

Various interacting mechanisms have been determined so far to take part in the pathophysiology of the development of anaemia in HF patients and it has been indicated that more than one mechanism usually serves to impair the homoeostasis of haemoglobin. These main contributing mechanisms, however, could be different from one patient to another^11^.

Dysregulation of the iron metabolism and homoeostasis system and the development of iron deficiency are among the most commonly seen mechanisms in patients with HF. Iron deficiency, even in the absence of anaemia, has been shown to be associated with poorer outcomes in these patients and to be even more prevalent than anaemia^20^. Absolute iron deficiency which occurs as the body’s iron stores become depleted, could originate from decreased content of this micronutrient in the diet of patients with HF, its low bioavailability or food restriction secondary to their respiratory symptoms, fatigue, nausea, decreased appetite, etc.^21,22^. Malabsorption of iron as a result of alteration in the normal physiology of the intestine and blood loss, mainly from the gastrointestinal tract, could proceed with the process of depletion of the body^23–25^. Defects in the utilization of iron ions, also known as functional iron deficiency hinder erythropoiesis and could lead to anaemia. This type of iron deficiency is induced following an increase in the cascade of inflammatory responses which mostly is mediated through hepcidin molecules. Hepcidin comes from hepatocytes where it serves as the master regulator of iron which acts by its interactions with cellular iron exporters, ferroportin^26,27^. The increased inhibitory effects of this regulator molecule upon inflammatory responses result in the failure of iron ions to leave the stores and be released into the extracellular fluid, “the functional pool”^26^. Besides, increased levels of hepcidin also deteriorate malabsorption by inhibiting ferroportins in the digestive system. This failed iron absorption and increased iron trapping limit the iron required for the erythroblast development. Furthermore, hepcidin inhibits the proliferation and survival of erythroblasts^28–30^.

Another important mechanism is the presence of inflammation. Inflammation and its associated signalling pathways have a mutual relationship with HF and they could both reinforce the other^31^. Studies have shown that as inflammation could be associated with the development and further progression of HF; the failing heart, ventricular dysfunction, and subsequent decreased flow of the vessels, themselves also aggravate the inflammatory responses by different direct and indirect means^31–33^. The level of proinflammatory cytokines such as interleukin (IL)-1, IL-6, and tumour necrosis factor- (TNF-) α, therefore, have been indicated to be higher in these patients with HF^34–36^. Either way, the pre-existing inflammation or HF-induced inflammation could result in anaemia in several ways. The level of IL-6 was measured in 2329 patients of the BIOSTAT-CHF cohort, revealing that more than half of them had higher plasma levels of this IL^35^. This high level of IL-6 stimulates the expression and transcription of Hepcidin through activation and phosphorylation of proteins of signal transducers and activators of the transcription family which results in both absolute and functional iron deficiency, as mentioned above^37^. This cytokine coupled with other proinflammatory cytokines, in particular TNF-α, affects the erythropoiesis negatively by downregulating the production of erythropoietin and dampening the response of its target cells, the progenitor cells, both mitigating the so-called compensatory hypoxia-induced production of erythropoietin^38,39^. These negative effects could be even intensified considering the high prevalence of renal disease in HF patients and the associated kidney-intrinsic impaired erythropoietin production and decreased hepcidin excretion^40^.

The occurrence of hemodilution is another contributory mechanism. The overarching problem of ventricular dysfunction with reduced arterial blood flow results in hypoperfusion of the kidneys and upregulation of the function of the renin-angiotensin-aldosterone system (RAAS). In the kidneys, macula densa senses the lack of sodium and signals the juxtaglomerular cells to secret renin^41,42^. Renin hydrolyzes angiotensinogen into angiotensin I which further undergoes another cleavage by angiotensin-converting enzyme (ACE). Angiotensin II, then, stimulates the adrenal glands and results in the release of aldosterone which acts on the distal tubule and collecting ducts to reabsorb sodium and water. This leads to hemodilution with normal absolute red cell count but decreased level of haemoglobin; this phenomenon is called pseudo-anaemia^43^. In a study on 37 patients with concomitant CHF and anaemia, red cell and plasma volume were measured using radioactive iodine-tagged albumin^44^. The study showed that hemodilution was present in 17 individuals (46%). Therefore, fluid retention and hemodilution could also contribute to the clinical profile of anaemia. There exist also other mechanisms that could be involved in the process of development of anaemia such as other nutritional deficiencies, folate, or vitamin B12^45^; however, they are less commonly seen.

## Medications used in patients with HF

The latest guideline for the management of patients with HF prepared by members of American Heart Association, American College of Cardiology and Heart Failure Society of America, 2022 AHA/ACC/HFSA Heart Failure Guideline, recommends 4 pharmacological treatment pillars for HF with reduced ejection fraction (HFrEF): medications inhibiting the renin-angiotensin system, ACE inhibitor (ACEi), angiotensin receptor blocker (ARB) or angiotensin receptor/neprilysin inhibitor (ARNi), beta-blocker (BB), mineralocorticoid receptor antagonist (MRA), and sodium-glucose cotransporter (SGLT) 2 inhibitor (SGLT2i)^46^. There is also a strong body of evidence regarding the use of a combination of hydralazine-isosorbide dinitrate in selected groups of HF patients. Additional medical therapies, particularly for patients with symptomatic HF include diuretics, soluble guanylate cyclase stimulators, and hyperpolarization-activated cyclic nucleotide-gated channel blockers. For patients with HF with preserved ejection fraction (HFpEF), medical management includes SGLT2i as the primary treatment, diuretics to relieve the symptoms of the patients and reduce congestion, or RAAS antagonists for control of blood pressure, if needed.

In a study on patients referring to outpatient cardiology clinics with HF, it was observed that ACEi/ARB was the most commonly used medication in these patients (79.4%), followed by BB (67.6%) and MRA (15.1%)^47^. These medications, especially the first four pillars, provide incremental benefits for reducing all-cause mortality, cardiovascular mortality, all-cause hospitalization, and hospitalization for HF^48^. In a population-based study on 2 132 800 patients with HFrEF and a New York Heart Association (NYHA) class of II–IV, it was estimated that the use of these four guideline-directed medications was associated with a 73% cumulative reduction in the relative risk of the all-cause mortality rate of them^49^. Some of these medications, however, could affect the erythropoiesis negatively. In this section, we aimed to mainly discuss the impact of these four pharmacological treatment pillars on haemoglobin concentration and pathophysiologic events that could be responsible for the observed impact.

### Beta blockers

Only three BBs have been recommended to be used in patients with HFrEF which are carvedilol (whether immediate-release or controlled-release), bisoprolol, and sustained-release metoprolol succinate. There are inconsistent findings regarding the effect of this drug class on haemoglobin concentration. In double-blinded Carvedilol or Metoprolol European Trial (COMET), either carvedilol or metoprolol tartrate was randomly administered to 3029 patients with HFrEF for a mean duration of 58 months^50^. In this study, it was seen that treatment with carvedilol contrary to metoprolol was associated with significant decreases in the level of haemoglobin by 0.2 g/dl after one year and by 0.4 after five in the enroled patients; Besides, the rate of development of anaemia in the patients without anaemia at the baseline of the study was significantly higher in the patients receiving carvedilol compared to those on metoprolol (with a relative risk of 1.22 with 95% CI of 1.05–1.41). It also has been mentioned in this article that a similar effect of carvedilol on haemoglobin concentration was observed in unpublished data from COPERNICUS and CHRISTMAS trials which both were double-blinded placebo-controlled randomized clinical trials (RCTs) on 2289 and 387 patients with HF with mean follow-up duration of about 10 and 6 months^50–52^.

However, contrary results have also been demonstrated^53–58^. In a 24-week RCT comparing the administration of carvedilol and bisoprolol to 84 patients with CHF, the level of haemoglobin was unaffected in either arm^53^. Likewise, in a 6-month cross-over randomized study, 70 patients with CHF were assigned to have a 2-month treatment with each of carvedilol, bisoprolol, and nebivolol^54^; the study showed no significant changes in haemoglobin level with these BBs. Khan *et al.* ^57^ study showed a beneficial effect of the initiation of BB on haemoglobin levels in CHF patients. In another trial, J-CHF Study, on HFrEF patients which lasted over a year, a paradoxical effect was seen: haemoglobin level significantly increased in 70 anaemic patients at baseline and significantly decreased in 290 non-anaemic patients^58^.

Production and release of erythropoietin from interstitial fibroblasts in the kidneys and proliferation of erythroid progenitor cells and therefore, the response induced by secreted erythropoietin molecules, both processes are increased by activations of the sympathetic nervous system^59,60^; treatment with BBs could decrease erythropoiesis through this mechanism. Besides, carvedilol is a non-selective BB with α_1_-blocking properties; therefore, compared to cardioselective BBs such as metoprolol which only block β_1_-adrenergic receptors, it could result in vasodilation, plasma volume expansion, and hemodilution, a phenomenon which could partly explain the findings of COMET study^50,61,62^. On the other hand, it could also be presumed that BBs may mitigate the vicious cycle of pathophysiologic processes involved in HF by improvements in heart function and exerting beneficial properties such as anti-inflammatory or antioxidant ones and therefore, reversing the pathologic mechanisms leading to anaemia^63–66^. By the way, even if treatment with BBs results in anaemia, the COMET study showed that carvedilol was not associated with severe anaemia and its overall positive effects outweigh this potential harm since a 17% reduction in mortality rate was found for the patients who received carvedilol in comparison to the patients in the other arm.

### Angiotensin-Converting enzyme inhibitors/angiotensin receptor blockers

The approved ACEis for treatment of HF are captopril, enalapril, fosinopril, lisinopril, perindopril, quinapril, ramipril and trandolapril and the approved ARBs are candesartan, losartan and valsartan^46^. The effects of these two medication classes on the level of haemoglobin/haematocrit are among the well-studied ones. In a systematic review and meta-analysis, the risk of development of anaemia in patients, not necessarily patients with HF, who were taking ACEi/ARB was measured^67^. In this meta-analysis, 7 studies comprising 29 061 patients were included and it was indicated that the use of ACEis and ARBs was associated with a relative risk of 1.56 (95% CI of 1.40–1.73) and 1.60 (95% CI of 1.27–2.00) for the development of anaemia, respectively. In studies conducted among those with heart failure, a similar effect was seen. In a secondary analysis of 4174 HF participants of the placebo-controlled SOLVD trial, it was reported that both groups of patients with and without prevalent anaemia on enalapril had lower levels of haematocrit after one year of the trial compared to patients in the placebo group^68^. Moreover, the odds ratio of incident anaemia was higher in the enalapril group (1.56% with a 95% CI of 1.26–1.93). However, despite the more than one-hundred percent increase in the hazard ratio of all-cause mortality in those with incident anaemia, the study showed that the use of enalapril was still associated with higher survival benefits. In another trial in 5010 patients with HF, Val-HeFT, valsartan use was associated with a mean change of about 0.4 g/dl in haemoglobin level compared to the placebo group by the fourth month of the trial and the mean haemoglobin levels of both arms remained stable over the following 20 months^69^.

Inhibition of the renin-angiotensin system could contribute to the development of anaemia through several means. First, angiotensin II as the main product of the renin-angiotensin system increases the production of erythropoietin. Angiotensin II decreases the oxygen tension within the kidney tissue by simultaneously reducing the oxygen supply as the result of constriction of efferent arterioles and arterioles entering the medulla and increasing the consumption of oxygen per each unit of blood flow^70^. In addition to this hypoxia-induced production of erythropoietin, studies have shown that angiotensin II could directly affect the expression of erythropoietin through angiotensin II receptor type 1; this binding results in phosphorylation and activation of mitogen-activated protein kinase family proteins and thereby, early growth response gene-1 transcription factor which binds to the promoter of erythropoietin gene^71^. Second, angiotensin II increases the proliferation of early erythroid progenitor cells^72^. Besides, in an experimental study on mice, it was shown that treatment with angiotensin II was significantly associated with decreased messenger ribonucleic acid (RNA) and serum levels of hepcidin and increased expression of divalent metal transporter-1 and ferroportin in duodenal tissues^73^; both of these effects were later reversed by olmesartan, an ARB. Furthermore, it has been reported that levels of insulin-like growth factor-1 and interleukin-12 were significantly lower in patients on renin-angiotensin inhibitors and N-acetyl-seryl-aspartyl-lysyl-proline level was higher in these patients; the first two are hematopoiesis stimulators and the last is an inhibitor of hematopoiesis^74–76^. Therefore, inhibition of the renin-angiotensin system with the use of these medication classes could indirectly lead to anaemia through upregulation of stimulators and downregulation of inhibitors of hematopoiesis^77^.

### Angiotensin receptor/neprilysin inhibitors

Inhibition of the renin-angiotensin system remains a mainstay for the treatment of patients with HFrEF over the past three decades; however, more recently, it has been recommended by guideline that treatment for those who tolerate an ACEi or ARB should be switched to an ARNi as it was associated with more favourable outcomes; besides, an ACEi or ARB should only be used when the treatment with ARNi is not feasible^46^. ARNi could also be used for control of blood pressure in patients with HFpEF if warranted with exerting greater effects in those with a lower left ventricle ejection fraction (EF). The addition of a neprilysin inhibitor to an ARB prevents neprilysin from degrading some cardiac and non-cardiac molecules such as natriuretic peptide (NP), bradykinin, substance P, angiotensin I, glucagonlike peptide-1, etc.^78^. Therefore, ARNi counteracts the maladaptive responses of both the RAAS and NP system in patients with HF resulting in vasodilation, increased blood flow to the kidneys, natriuresis, and reduced retention^79–81^.

Although there is limited evidence regarding the relationship between ARNi use and haemoglobin concentration, the literature suggests that similar to other medications inhibiting the renin-angiotensin system, ARNi could join other existing factors in HF patients for the development of anaemia. The proposed mechanism of this relationship is mainly attributable to its ARB component; it seems that usage of sacubitril alone, however, might ameliorate anaemia and increase the haemoglobin concentration by elevation of the level of angiotensin I and thereby angiotensin II and its downstream pathway and by decreasing volume retention. However, the use of sacubitril alone is not recommended in treatment for HF^82,83^. Sacubitril/Valsartan is the sole available ARNI on the market. In the largest RCT conducted to assess the efficacy and safety of ARNi, PARADIGM-HF, sacubitril/valsartan and enalapril were administered to 8442 patients with HFrEF^84^. In this paradigm-shifting trial, it was observed that a comparable percentage of both arms, for both 5%, had more than a 20% decrease in their level of haemoglobin/haematocrit^85^. In another retrospective cohort study by Yang *et al.* ^86^, in more than one-third of patients with HF for whom sacubitril/valsartan was initiated, a decreasing pattern of haemoglobin concentration over 12 months was seen which was associated with a higher hazard ratio of mortality. Another important result of this study was that there was not a significant difference between survival rates of patients with haemoglobin levels of less or above 12 g/dl among those who were receiving treatment for anaemia whereas for patients who were not on any medications for anaemia, level of haemoglobin was associated with mortality rate; this highlights the importance of detection of treatment of anaemia as a comorbidity while trying to optimize the treatment for HF.

Inconsistently, in a study on 39 patients with HFrEF who had developed cardiorenal syndrome, the mean haemoglobin level significantly increased by 0.7 g/dl, and the prevalence of anaemia significantly decreased from 64.7% to 38.4% following three-month after initiation of sacubitril/valsartan^87^. However, this finding should be approached with caution as the study lacks a control arm and was conducted on a small population with a short duration of observation who was also afflicted with cardiorenal syndrome which may benefit more from neprilysin inhibitors; furthermore, anaemic patients at the baseline received treatment for anaemia, that is iron supplements/ erythropoiesis-stimulating agents (ESA).

### Mineralocorticoid receptor antagonists

The dynamics of the effect of MRAs on haemoglobin concentration in human participants has not been reported; however, in the study results section found at ClinicalTrials.gov of double-blind, placebo-controlled RCTs such as TOPCAT trial (NCT00094302) conducted on 3445 patients with HFpEF assessing the efficacy and safety of spironolactone with a median follow-up of about 3.3 years, EMPHASIS-HF trial (NCT00232180) on 2737 patients with HFrEF receiving eplerenone for a median follow-up of 1.75 years, J-EMPHASIS-HF trial (NCT01115855) on 221 patients with HFrEF receiving eplerenone for a median follow-up of near to three years and ARTS-HF trial (NCT01807221) of finerenone versus eplerenone on 1066 patients with worsening HFrEF with concurrent type two diabetes mellitus (DM) and/or chronic kidney disease, the rate of development of anaemia was not significantly higher in those in MRA arm^88–91^. In a case series of four anaemic patients with primary glomerulonephropathy, ACEi/ARB was presumed to be the root for the low haemoglobin concentration and was then discontinued and patients were started on MRAs; this switch led to the resolution of the anaemia^92^.

So far, little has been known regarding the relationship between MRAs, spironolactone and eplerenone, and the mechanisms involved in the pathophysiology of anaemia. In a study by Mleczko-Sanecka and colleagues, they reevaluated a comprehensive list of hepatic hepcidin regulators identified in their previous work through genome-wide RNA interface screen to limit the regulators to hits that were capable of being aimed to direct and specific pharmacological manipulation^93,94^. Later, by use of small interfering RNAs, the effectiveness of knock-down of the hit genes in alteration of the hepcidin messenger RNA levels was validated. Thereafter, small-molecule testing was conducted to identify the appropriate drug. In this study, it was shown that aldosterone antagonists, spironolactone and eplerenone, were both associated with decreased levels of messenger RNA level of hepcidin in primary human liver cells in a dose-dependent manner. Besides, 2-week administration of spironolactone to mice led to a significant reduction in the expression and plasma concentration of hepcidin. On the other side, in another experimental trial, aldosterone and fludrocortisone, aldosterone’s analogue, significantly increased both transcription and translation of the erythropoietin gene^95^. Spironolactone and eplerenone target aldosterone receptors and therefore, have no other inhibitory effect on other components of the RAAS system and angiotensin II-activated pathways. Besides, the level of angiotensin II may even rise with the use of this medication class due to a feedback effect. Therefore, it seems that the use of MRAs could ameliorate anaemia through suppression of hepcidin and may also result in decreased secretion of erythropoietin. The net effect of this medication class on the concentration of haemoglobin, whether these results could be valid in humans, whether there be other mechanisms, etc. are the gaps in this scenario that remain to be filled with further studies.

### Sodium-Glucose cotransporter 2 inhibitors

Canagliflozin, empagliflozin, dapagliflozin, and ertugliflozin are the among SLGT2is approved by the United States Food and Drug Administration (FDA) to improve the control of glycemic indices in adult patients with type 2 DM (T2DM). Besides, it has been approved by the FDA and recommended by the latest guideline to administer dapagliflozin for all patients with HFrEF or empagliflozin for patients with HFrEF and HFpEF, both, without consideration of baseline diabetes status, unless contraindicated or not tolerated^46,96^. The use of canagliflozin, however, is mainly recommended for those HF patients with diabetes-induced albuminuria^97^. Sotagliflozin, a dual inhibitor of SGLT1 and SGLT2 proteins, has also recently been shown to have a promising effect in patients with HF. In SOLOIST-WHF RCT which was conducted on 1222 patients with T2DM who had recently been hospitalized for worsening condition of their failing heart, the patients were randomized to take either sotagliflozin or placebo^98^. In this study, it was demonstrated that following a median of 9 months, sotagliflozin was associated with significantly reduced hazards of the combined endpoint of death from cardiovascular causes and hospitalizations and urgent visits for HF by 33% (95% CI of −0.48 to −0.15). Sotagliflozin has been approved by the European Medicines Agency to be used as an adjunct to insulin to optimize the blood glucose level in patients with type 1 DM and uncontrolled glycemic indices and a body mass index of at least 27 kg/m^2^ ^99^. Although the FDA had issued a complete response letter regarding the use of sotagliflozin for the treatment of adults with type 1 DM in 2019 and has not approved any use of this medication as of 2022, it has accepted to review New Drug Application for sotagliflozin for patients HF in May 2023^100^.

There is a strong body of evidence regarding the effect of each of SGLT2is on the level of haematocrit/haemoglobin. In the placebo-controlled EMPA-REG OUTCOME study assessing the effects of empagliflozin on patients with T2DM and established cardiovascular disease, 7020 patients were enroled^101^. The study showed that empagliflozin at a dose of 10 mg and 25 mg per day was associated with a 4.8% (± 5.5) and 5.0% (± 5.3) increase in haematocrit level and 0.8 g/dl (± 1.3) and 0.8 g/dl (± 1.3) increase in haemoglobin level, respectively, compared to change of 0.9% (± 4.7) and −0.1 g/dl (± 1.2) in haematocrit and haemoglobin level of placebo arm. Consistently, both EMPEROR-Reduced and EMPEROR-Preserved trials showed a similar positive effect in the level of haematocrit in those receiving empagliflozin^102,103^; besides, EMPEROR-Reduced reported that the incidence of new-onset anaemia over a median of 16 months was statistically significantly lower among the patients receiving empagliflozin with reduced hazards of −0.51 (95% CI of −0.59 to −0.41)^104^. These beneficial effects were also seen with dapagliflozin and canagliflozin. In post hoc analysis of data from 14 placebo-controlled RCTs with a population of 5325 patients with T2DM who were started on dapagliflozin, the change in haematocrit/haemoglobin was significant in the dapagliflozin-treated group^105^. In this study, it was also shown that dapagliflozin was associated with a reduced rate of new-onset anaemia and an increased rate of corrected anaemia in those on dapagliflozin by week 24. Placebo-controlled trials of DAHA-HF on 4744 HFrEF patients and CANVAS on 10142 T2DM patients with high cardiovascular risk and CREDENCE on 4401 patients with concurrent T2DM and chronic kidney disease showed similar findings regarding the effect of dapagliflozin and canagliflozin, respectively^106–108^.

Besides, studies have also indicated that this positive impact of SGLT2 is on haemoglobin levels is one of the main mechanisms by which this medication class exerts beneficial effects. In a secondary analysis of the EMPA-REG OUTCOME Trial, mediators contributing to the reduction observed in mortality due to cardiovascular causes with the use of empagliflozin were explored^109^. In this study, change in haematocrit/haemoglobin was reported to be the strongest variable with a proportion mediated of 51.8% of the reduction. In another study using data from the CANVAS trial, similarly, change in haemoglobin level was among the key three mediators of effects of canagliflozin on HF with a proportion of about 50% whether when measuring changes in the early phase of the study or when considering average levels within the long-term follow-up^107^.

SGLT2is could result in a marked increase in the level of haemoglobin/haematocrit within the first weeks of its use; this increase has been mainly attributed to its diuretic effect of SGLT2is^96^. SGLT2 proteins are responsible for the reabsorption of the majority of the filtered glucose. By inhibition of these proteins, both glucose and sodium reabsorption are impaired, leading to diuresis, plasma volume contraction, and thereby, hemoconcentration which is in line with changes in other makers of plasma volume such as an increase in albumin and total protein level. The increase in haemoglobin/haematocrit level beyond the first weeks without further increase in albumin level and the persistence of this effect over years of administration along with the proportionally larger effects of SGLT2is on haemoglobin/haematocrit compared to albumin level, however, suggest that other mechanisms are also involved^105,108^. Studies have shown that SGLT2is could also ameliorate anaemia by enhancing the process of erythropoiesis. SGLT2is activates energy deprivation sensors such as sirtuin-1 and hypoxia-inducible factor-2α and their downstream signalling pathway which could give rise to increased synthesis of erythropoietin^96,110^. Besides, hypoxia-inducible factor-1α, the other isoform, also rises in response to decreased medullary oxygen tension and upregulation of antidiuretic hormone which themselves result from inhibition of SGLT2 proteins^111,112^. Furthermore, SGLT2is could reduce the glucose content within the tubulointerstitial tissue by blocking the actions of SGLT2s and thereby lower the unfavourable changes that may be induced by glucose which is more marked in diabetic patients such as inflammation, oxidative stress, and fibrosis^113–115^. This renoprotective effect coupled with the mechanisms upregulating hypoxia-induced factors augment the erythropoiesis. Beyond these effects, SGLT2is possesses anti-inflammatory properties and suppresses hepcidin and thereby, maintains the bioavailability of iron for progenitor cells^116,117^.

## Limitations

Our study provided a comprehensive review of the existing literature regarding the impact of the four main pharmacological pillars recommended by the current guidelines on the level of haemoglobin and/or the development of anaemia in patients with HF with regard to the potential pathophysiologic mechanisms. The study has, however, several limitations. A notable portion of the literature addressing the impact of these medications on the haemoglobin level is derived from observational studies, which carry a lower level of evidence. Furthermore, in some studies, the impact on haemoglobin levels or the development of anaemia was not the primary outcome, leading to a lack of additional statistical adjustments beyond randomization, if any randomization was conducted, to account for confounding factors. Additionally, there were variations in the definition of anaemia, dosage, and the type of intervention across the studies which introduced heterogeneity to the current literature. Fourth, some important published RCTs lacked data on changes in the haemoglobin level following treatment with these medications, even though this outcome was specified as an outcome measure of interest in the studies’ protocol. Lastly, there are conflicting findings regarding the effects of certain medications in the literature, and the sample sizes for some medications were insufficient to draw solid conclusions. These aspects underscore the need for further studies with a higher level of evidence.

## Conclusion

Although HF has an intricate and multifactorial nature in patients with HF, predominantly arising from the pathologic conditions inherent to the disease, the reviewed literature indicates that medications recommended by guidelines and commonly prescribed for these patients may play a role in the development of anaemia. Literature suggests that BBs and ACEis/ARBs/ARNis result in decreased levels of haemoglobin whereas SGLT2is augment the process of erythropoiesis and leads to the resolution of anaemia. The robust body of evidence for ACEis/ARBs and SGLT2is contrasts with limited studies on BBs and MRAs which reveals the need for more studies on these medication classes. Importantly, even medications that may induce or exacerbate pre-existing anaemia in patients with HF demonstrate overall favourable outcomes compared to placebos, emphasizing the net positive effects of the treatment with these pillars. Besides, it was shown that among patients who were receiving medications with negative effects on the level of haemoglobin, there was no difference in the rate of mortality between anaemic and non-anaemic patients when both were on treatment for anaemia whereas for patients who were not on any medications for anaemia, level of haemoglobin was associated with mortality rate. Our findings underscore the significance of detecting and treating anaemia in HF patients, prompting physicians to carefully weigh the potential negative impacts of these essential HF medications and monitor patients to avert unfavourable outcomes. Further research is, therefore, warranted to explore this relationship, particularly when comes to BBs and MRAs. Additionally, there is a need to assess whether implementing additional strategies, such as routine complete blood count checks or initiating antianemic medications at a higher haemoglobin threshold, is helpful. Furthermore, risk stratification of patients with HF regarding the development of anaemia after initiating these medications could be considered as a potential measure to mitigate the risk of anaemia in this population.

## Ethical approval

Not applicable. Our study is a review and does not involve patients.

## Consent

Not applicable. Our study is a review and does not involve patients.

## Source of funding

This study was not supported by any sponsor or funder.

## Author contributions

Conceptualization: D.H., E.T., and T.H.A.; Investigation: D.H., E.T., and T.H.A.; Writing—original draft: D.H and E.T.; Writing—review and editing: D.H., E.T., and T.H.A; all authors gave final approval of this version to be published, and are fully accountable for the content of the manuscript.

## Conflicts of interest disclosure

The authors have no conflicts of interest to declare.

## Research registration unique identifying number (UIN)

Not applicable.

## Guarantor

The corresponding author, Erfan Taherifard, accept full responsibility for the work and/or the conduct of the study, had access to the data, and controlled the decision to publish.

## Data availability statement

All data generated or analysed during this study are included in this manuscript.

## Provenance and peer review

No; our paper was not invited.
